# Development of titanium 3D mesh interlayer for enhancing the electrochemical performance of zinc–bromine flow battery

**DOI:** 10.1038/s41598-021-83347-1

**Published:** 2021-02-24

**Authors:** Je-Nam Lee, Eunbyul Do, Youngkwon Kim, Ji-Sang Yu, Ki Jae Kim

**Affiliations:** 1 Advanced Batteries Research Center, Korea Electronics Technology Institute (KETI), #25, Saenari-ro, Bundang-gu, Seongnam-si, Gyeonggi-do 13509 Republic of Korea; 2 Department of Energy Engineering, Konkuk University, Neungdong-ro 120, Gwangjin-gu, Seoul, 05029 Republic of Korea

**Keywords:** Batteries, Energy storage

## Abstract

Zinc dendrite growth negatively affects zinc–bromine flow battery (ZBB) performance by causing membrane damage, inducing self-discharge. Herein, in a ZBB, a conventional polymer mesh was replaced with a titanium-based mesh interlayer; this provided additional abundant active sites for the Zn^2+^/Zn redox reaction and well-developed electrolyte flow channels, which resulted in improved reaction kinetics and suppressed Zn dendrite growth. Compared with a ZBB cell comprising a conventional polymer mesh and a carbon-based electrode, the ZBB cell using the titanium mesh interlayer and a carbon-based electrode showed significantly reduced frequency of the refreshing process, which occurs at regular cycling intervals during practical use for removing residual zinc dendrites in ZBB; also, the average energy efficiency at a current density of 40 mA cm^−2^ increased by 38.5%. Moreover, the modified ZBB cell exhibited higher energy efficiency at a high current density of 80 mA cm^−2^, which is an improvement of 14.7% than in case of the contemporary polymer mesh. Consequently, this study can provide helpful insights for new anode side structures including spacer mesh for developing high-performance ZBBs.

## Introduction

The increasing deployment of intermittent renewable energy sources, such as solar and wind power, has increased the demand for large-scale electrical storage devices to improve grid reliability and power quality^1–7^. Redox flow batteries (RFBs) have been widely considered as the most compatible energy storage technology because of their simple design, good scalability, good cycle efficiency, and reliable long lifespan^8–10^. Among various RFBs, all-vanadium redox flow batteries (VRFBs) have developed enough to be commercialized because of their low ion contamination and high energy efficiency^11^. Recently, however, the high cost and limited reserves of vanadium raw materials and relatively low energy density have been barriers to the widespread commercialization of VRFBs. Consequently, the usage of low-cost active materials in RFBs is critical for achieving an economical RFB, leading to its successful commercialization.

Zinc–bromine flow batteries (ZBBs) have been considered as a promising alternative for large-scale energy storage because of the relatively high energy density due to the high solubility of Zn^2+^ and the abundance of zinc compounds, which also results in availability of low-cost materials^12,13^. Compared with the energy density of VRFBs (25–35 Wh kg^-1^)^11^, ZBBs show an energy density of ~ 65–75 Wh kg^−1^^5,14,15^. In addition, the material cost of ZBBs based on the system level is approximately $200 kWh^-1^, which is lower than that of VRFBs (~ $750 kWh^-1^) and even competitive to that of Li batteries ($350–550 kWh^-1^)^16,17^. Therefore, ZBBs can have tremendous potential to significantly reduce the cost of energy storage systems (ESSs). However ZBBs face several critical issues, such as poor kinetics of the Br_2_/Br^-^ redox reaction, toxic charge product Br_2_, severe self-discharge, and Zn dendrite growth during the charge and discharge process, which are possible barriers for the commercialization of ZBBs as ESSs^17–19^. Several studies have been attempted to develop a positive electrode for enhancing electrochemical performance or a new electrolyte additive for suppressing the severe self-discharge^20^. Interestingly, even though the performance of the ZBBs, such as cycle stability and energy efficiency, is affected by the Zn dendrite growth in the negative electrode, relatively less attention has been paid to the negative electrode. In the contemporary method suppress zinc dendrite growth during the cycles, an electrode refreshing process, involving an additional discharge process to wash away the residual zinc from the electrode surface, is performed at regular cycling intervals in practical use. However, the electrode refreshing process is slightly time consuming, and thus, leads to inevitable cost and time loss during ZBB operation. Therefore, in terms of the utility efficiency of ZBBs, it is necessary to reduce the time required for the electrode refreshing process or eliminate the process by modifying the electrode material and design in the negative side.

**Figure 1:**
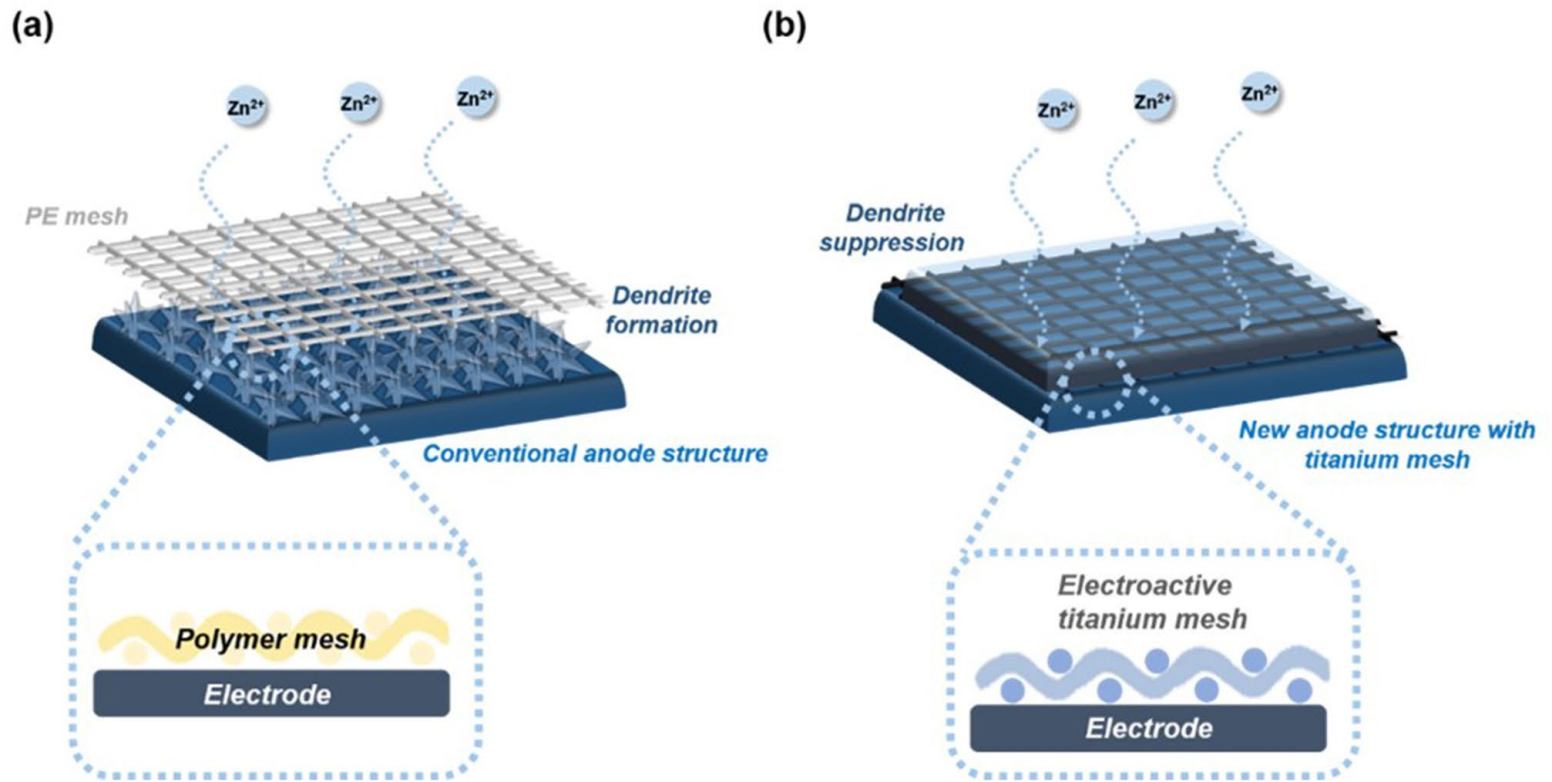
Schematic illustration of **(a)** typical anode structure (GE) and **(b)** new anode structure with electroactive titanium mesh (donated as 3D-Ti-GE anode).

Typically, a polymer mesh is placed as a spacer between a carbon-polymer electrode and separator to provide an electrolyte pathway and retain dimensional stability in practical use of ZBBs. However, the polymer mesh is not an electric conductor; therefore, its use results in reduced effective active area of the carbon-polymer electrode for zinc plating and stripping and limits the charging current density^21,22^. Based on literature survey, clearly, material design of the spacer in the negative side should be examined to ensure effective utilization of the carbon-composite electrode for Zn plating and stripping as well as suppression of Zn dendrite growth on the surface of the negative electrode. Thus, herein, we propose to replace the conventional polymer mesh with a titanium-based mesh to impart versatile functionality by providing additional electroactive area, retaining dimensional space upon deposition and stripping, and sufficient electrolyte diffusion at the anode side. By simply using a titanium spacer material instead of polymer, the proposed anode structure effectively alleviates zinc dendrite growth and enhances the kinetics of the anode material, facilitating good rate capability (Fig. 1).

## Results and discussion

This study aims to enhance the electrochemical performance of a carbon-polymer electrode by replacing a polymer mesh with a three-dimensional (3D) titanium mesh as a spacer. The proposed 3D titanium mesh can provide additional active sites for zinc plating and uniform current distribution on the electrode surface for suppression of Zn dendrite growth.

**Figure 2:**
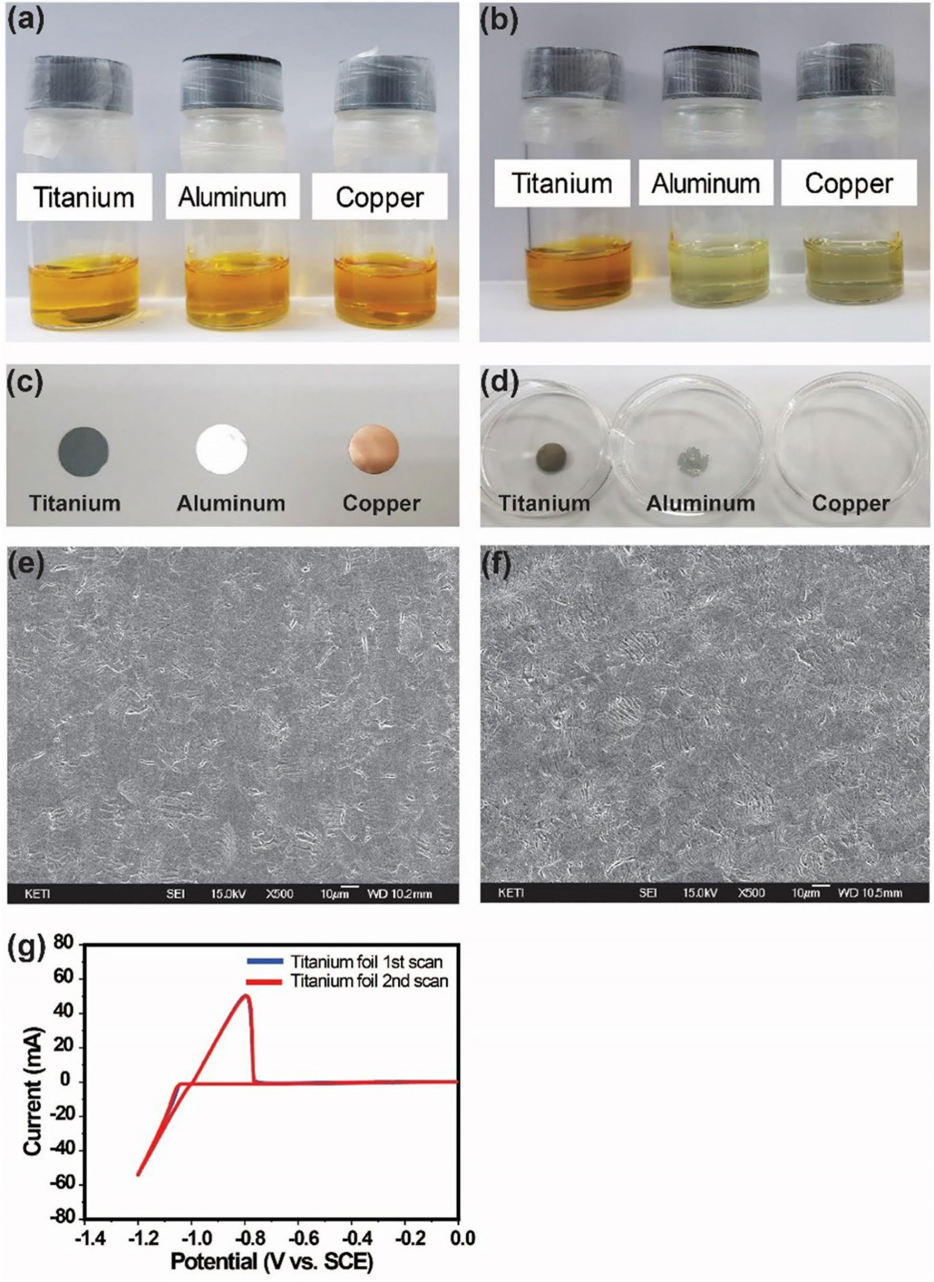
Photographs of ZBB electrolyte with different metal foils (**a**) before storage and (**b**) after 80 h storage. Photographs of metal foils (**c**) before electrolyte storage and (**d**) after 80 h electrolyte storage. SEM images of (**e**) pristine titanium foil and (**f**) stored titanium foil in the electrolyte. (**g**) Cyclic voltammogram (CV) obtained from titanium foil electrode with 2.25 M ZnBr_2_ solution.

To clearly understand whether titanium can be used as a spacer in ZBBs, storage test was performed, and the results are shown in Fig. 2. For comparison, aluminum (Al) and copper (Cu) foils were stored in the same electrolyte. After 80 h storage, severe corrosion is observed in the ZBB electrolyte containing aluminum and copper (see Fig. 2a,b), while there is no significant change in the case of titanium (Fig. 2b,d). Additionally, Scanning Electron Microscopy (SEM) images of the pristine titanium mesh (Fig. 2e) and stored titanium mesh (Fig. 2f) show that there is no severe structural degradation of the titanium mesh in the electrolyte after 80 h storage. To further clarify the electrochemical reversibility of zinc zinc deposition/stripping behavior on the titanium metal, cyclic voltammogram (CV) profiles were obtained. Figure 2g shows the CV profile of the titanium foil at a scan rate of 20 mV s^−1^ in a solution containing 2.25 M ZnBr_2_, 3 M KCl as the supporting electrolyte, and 0.6 M *N*-ethyl-*N*-methylpyrrolidinium bromide (MEP) as the complexing agent. As might be expected, deposition potential and crossover potential at − 1.037 and − 1.001 V, respectively, corresponding to the nucleation are observed, along with an anodic peak at − 0.797 V corresponding to redox potentials of the Zn^2+^/Zn^23^. The observed redox potential and related nucleation overpotential (NOP), which is the voltage between deposition and crossover potential, are similar to those reported in other studies^23,24^. This implies that when used with a carbon-polymer negative electrode in the ZBB system, the 3D titanium mesh interlayer can provide active sites for reversible zinc deposition/stripping, and thus, can act as a spacer as well as an electroactive material.

**Figure 3:**
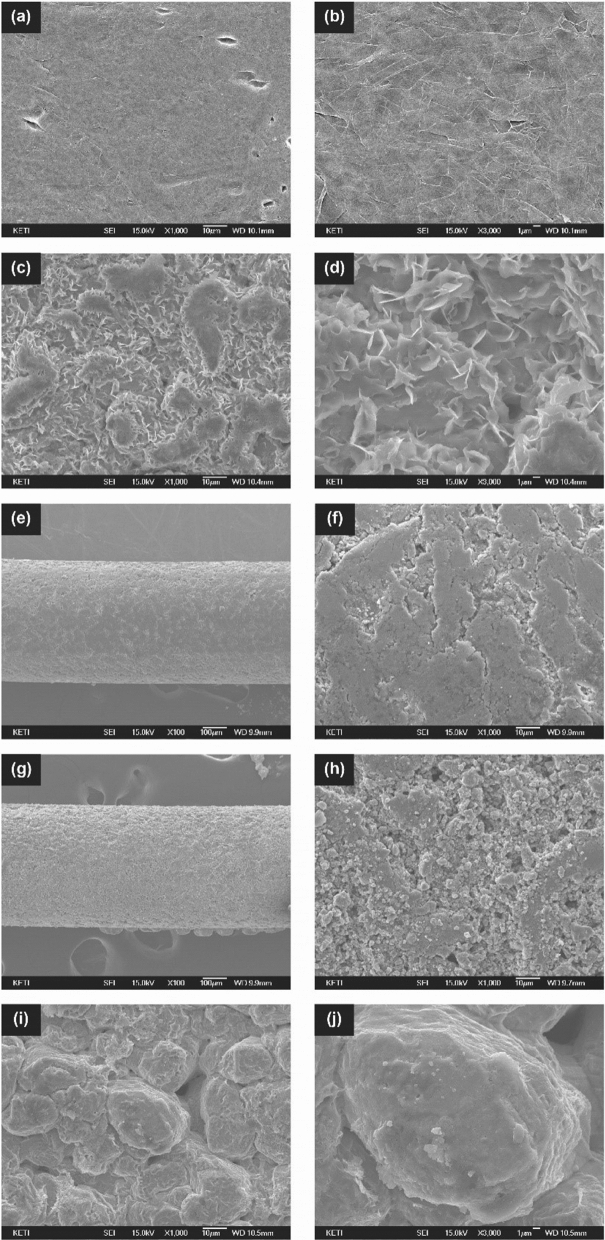
SEM images of (**a**,**b**) GE anode, (**c**,**d**) GE anode after initial zinc deposition. SEM images of (**e**,**f**) surface of titanium mesh interlayer, (**g**,**h**) surface of titanium mesh after initial zinc deposition and (**i**,**j**) 3D-Ti-GE below titanium mesh after initial zinc deposition.

To investigate the effect of the 3D titanium mesh on the electrodeposition behavior of zinc, a graphite plate electrode with the 3D titanium mesh (denoted as 3D-Ti-GE) was prepared and then zinc deposition behavior on the prepared electrode was investigated. For comparison, zinc deposition behavior of the graphite plate electrode without the 3D titanium mesh (denoted as GE) was also studied. Figure 3a–j depict the SEM images of the GE and 3D-Ti-GE. After initial deposition of zinc, significant zinc dendrites are clearly observed in case of the GE (Fig. 3c,d). On the other hand, zinc dendrites are not observed on the 3D-Ti-GE (Fig. 3i,j), being accompanied by that zinc with mostly particle like structures are appeared on the surface of both 3D-Ti-GE and titanium mesh (Fig. 3g,h).

**Figure 4:**
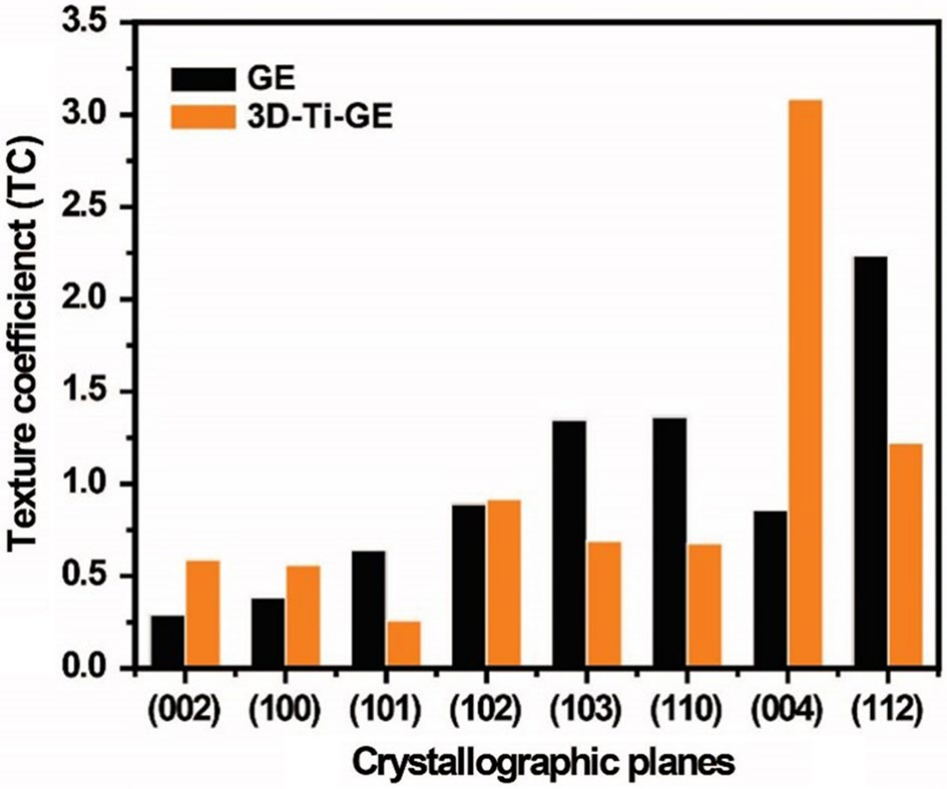
Texture coefficient (TC) values of the zinc crystallographic planes of deposited zinc from the GE and 3D-Ti-GE after charging.

The preferred orientation of zinc deposition on GE and 3D-Ti-GE was estimated by texture coefficients (TCs) obtained using Eq. (1). Figure 4 shows the XRD patterns of the zinc deposited after 30 min at a current density of 40 mA cm^−2^

1 $$ TC = \frac{{I_{{R\left( {hkl} \right)}} }}{{N^{ - 1} (\sum I_{{R\left( {hkl} \right)}} )}} $$

where *n* is the number of chosen planes corresponding to peaks from the X-ray diffraction (XRD) patterns. Eight planes were selected for TC comparison. *I*_*R*(hkl)_ is the ratio between the peak intensity of the plane (hkl) and the intensity of plane (hkl) in the standard zinc powder. In the control anode, the preferable planes are pyramidal (112) and (103) and prismatic (110)^25^. With the introduction of the titanium interlayer electrode structure, the planes (004), (112), and (102) appear as the main texture coefficients. Considering that TC > 1 indicates a preferred texture^26^, the favorable zinc growth plane is the (004) plane with the 3D-Ti-GE. This basal plane is parallel to the substrate surface^27^. In addition, the TC value of the basal (002) plane is higher for the 3D-Ti-GE. This indicates that the 3D-Ti-GE is an important factor in reducing dendrite growth on the anode.

**Figure 5:**
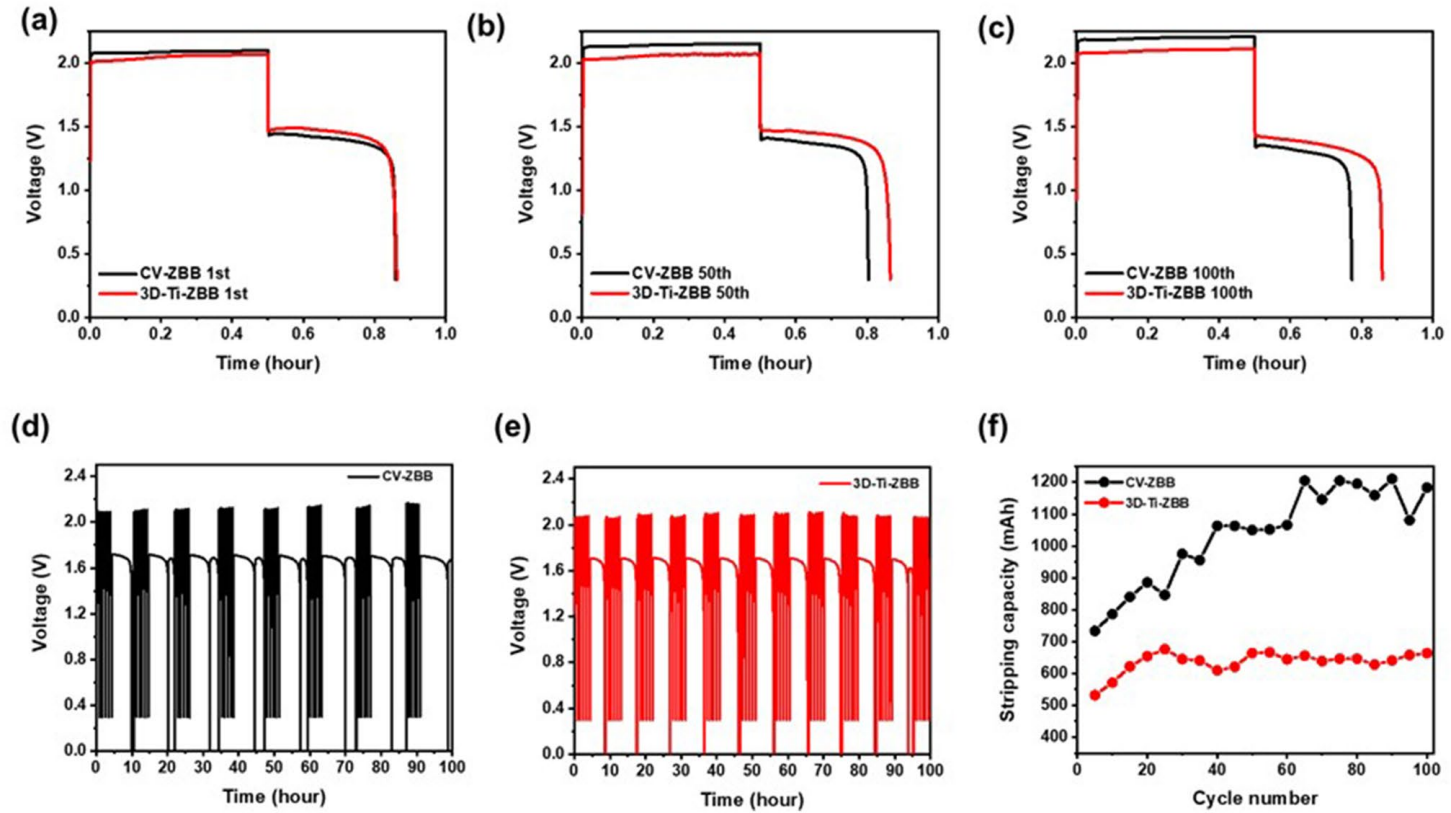
(**a**–**c**) Comparison of charge–discharge voltage profiles of CV-ZBB and 3D-Ti-ZBB single cells at 1st, 50th, and 100th cycles. Charge–discharge and stripping voltage profiles of ZBB single cells with (**d**) GE (ref) and (**e**) 3D-Ti-GE over 100 cycles. (**f**) Electrode refreshing capacities (zinc stripping capacities) of ZBB single cells with GE (ref) and 3D-Ti-GE for current densities from 5 to 1 mA cm^−2^ performed every five cycles over 100 cycles.

**Figure 6:**
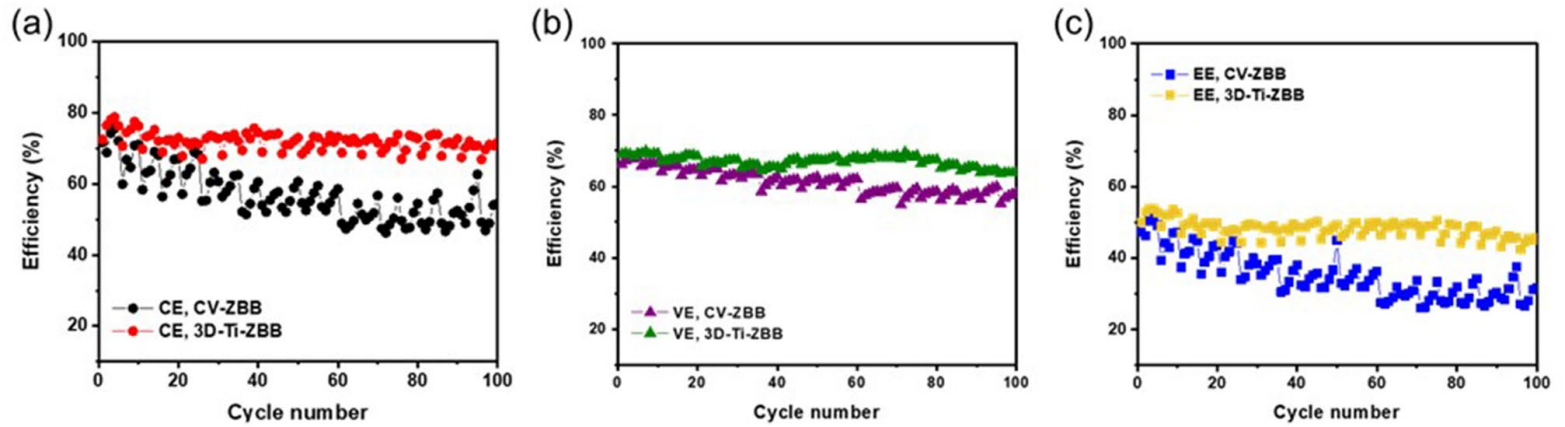
Comparison of (**a**) coulombic, (**b**) voltage, and (**c**) energy efficiencies of the CV-ZBB single cell and 3D-Ti-ZBB single cell.

To further understand the effect of the 3D-Ti-GE on the electrochemical performance of ZBBs, a ZBB cell with 3D-Ti-GE (denoted as 3D-Ti-ZBB cell) was galvanostatically charged and discharged at a constant current density of 40 mA cm^−2^. For comparison, a ZBB cell employing the conventional electrode (denoted as CV-ZBB cell) was also prepared. As shown in Fig. 5a, the initial charge–discharge profiles of CV-ZBB and 3D-Ti-ZBB cells are similar. However, during prolonged cycling, the 3D-Ti-ZBB cell shows a lower charge voltage and a greater retained discharge capacity than in case of the CV-ZBB cell (Fig. 5b,c), implying that the 3D-Ti-GE positively influences the electrochemical performance of ZBB. This is mainly attributed to the increased active area and enhanced kinetics of the anode structure, which facilitates the reversibility of zinc deposition/stripping behavior and decreases the inactive or dendritic zinc growth. Figure 5d,e depict successive charge–discharge and stripping profiles of the 3D-Ti-ZBB and CV-ZBB cells, respectively. During the same operation time, the 3D-Ti-ZBB cell undergoes more cycles than the CV-ZBB cell. The charge and discharge operation times do not differ significantly, which shows that introducing the 3D-Ti-GE reduces the time required for the electrode refreshing process. To clearly understand the inhibiting effect of 3D-Ti-GE on zinc dendrite growth, the zinc stripping capacities of the single unit cells obtained from the electrode refreshing process were compared. According to Fig. 5f, the stripping capacity of the CV-ZBB cell shows a gradual increase after every fifth cycle while that of the 3D-Ti-ZBB cell does not increase during cycling. This result indicates that the 3D-Ti-ZBB cell shows a 38.5% decrease in overall stripping capacity than that of the CV-ZBB cell at a current density of 40 mA cm^−2^. Figure 6a,b show the coulombic (CE) and voltage efficiencies (VE) of the 3D-Ti-ZBB and CV-ZBB cells. The 3D-Ti-ZBB cell exhibits a VE of 66.9% and a CE of 72.0% at a current density of 40 mA cm^-2^, which are higher than those of the CV-ZBB cell (VE = 61.2%, CE = 56.8%). Thus, the 3D-Ti-ZBB cell exhibited a high energy efficiency of 48.2%, which is approximately 1.38 times higher than that of the CV-ZBB cell (Fig. 6c). Thus, the outstanding electrochemical performance of the 3D-Ti-ZBB cell results from the 3D-Ti-GE facilitating the utilization of zinc deposited during charging, which reduces the residual zinc that may participate in mossy dendritic growth.

**Figure 7:**
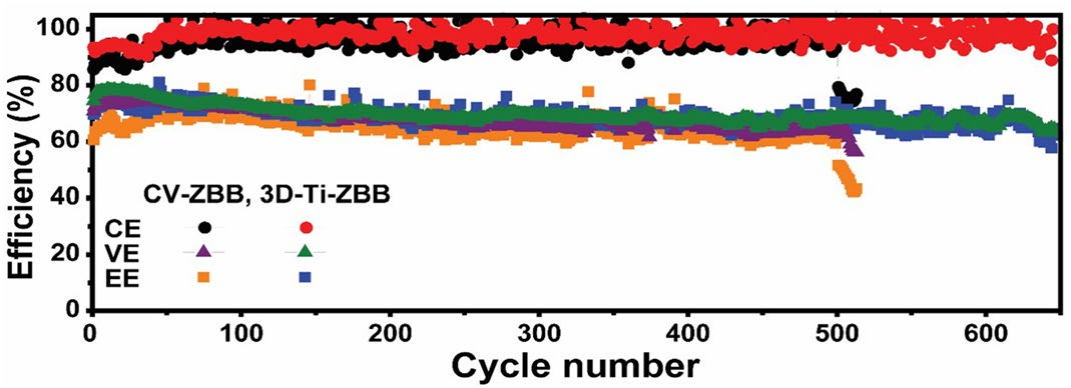
Comparison of coulombic, voltage, and energy efficiencies of ZBB single cells without the refreshing process during prolonged cycling.

When the refreshing process was not used, the electrochemical performances of the CV-ZBB cell and 3D-Ti-ZBB cell were monitored at a constant current density of 80 mA cm^-2^ for 7.5 min. As shown in Fig. 7, the CV-ZBB cell showed a rapid decrease in efficiency after 500 cycles. This is attributed to the accumulated residual zinc from the dendritic growth, which penetrates the membrane and induces cell failure. On the other hand, the 3D-Ti-ZBB cell retained decent cycling stability over 650 cycles. This implies that dendrite growth is effectively alleviated in the 3D-Ti-GE cell.

**Figure 8:**
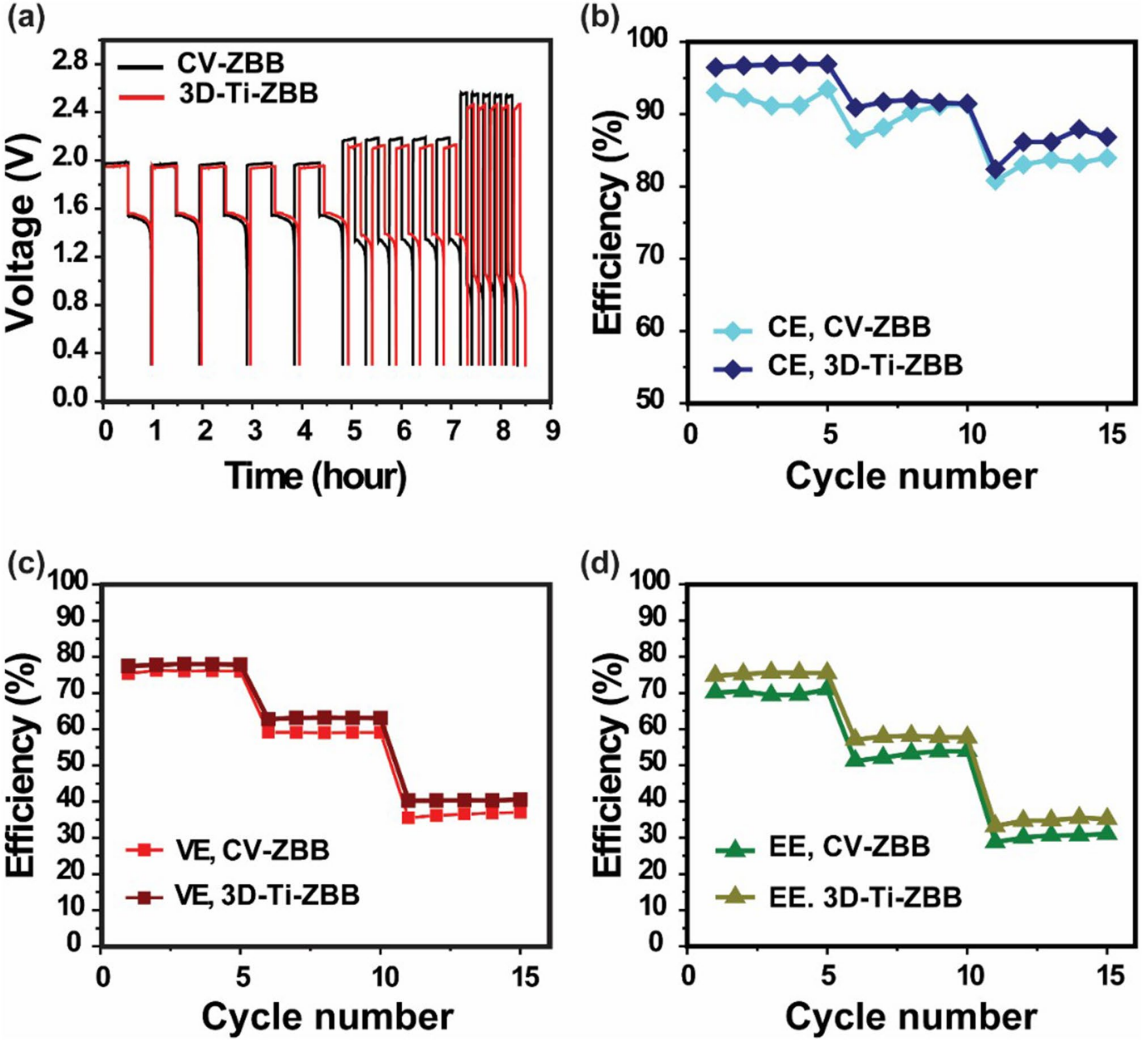
(**a**) Charge–discharge voltage profiles; (**b**) columbic, (**c**) voltage, and (**d**) energy efficiencies of ZBB single cells with GE and 3D-Ti-GE structure under various current densities.

The introduction of 3D-Ti-GE is expected to improve the rate capability because it increases the active area of the electrode. Moreover, the porous mesh structure might also support electrolyte diffusion^28^, thus improving reaction kinetics. Therefore, to explore the effect of 3D-Ti-GE on the charge–discharge rate capability, the cells were charged and discharged at currents rates between 20 and 80 mA cm^−2^. Figure 8a displays the charge–discharge profiles of the 3D-Ti-ZBB and CV-ZBB cells at various current densities. Irrespective of the current density, the 3D-Ti-ZBB cell shows a lower IR drop (111, 293, 585 mV at 20, 40, 80 mA cm^−2^ , respectively) than in the CV-ZBB cell (138, 347, 699 mV at 20, 40, 80 mA cm^−2^ , respectively), suggesting less electrochemical polarization due to the increased active area with lower concentration polarization due to the porous mesh interlayer. As a result, the cell employing 3D-Ti-GE exhibits higher VE (40.38%) and CE (85.9%) than those of the cell employing the conventional electrode (VE = 36.43%, CE = 82.96%) at a current density of 80 mA cm^−2^ (Fig. 8b–d). This improvement is ascribed to the improved kinetics on the anode and the reduced cell resistance.

## Conclusion

We investigated the effect of a 3D titanium mesh as a spacer in ZBBs to suppress zinc dendrite growth and enhance rate capability. The addition of the 3D-Ti-GE to the anode affected the anode kinetics and reversibility of zinc. Clearly, the 3D-Ti-GE can increase the active area of the anode, thereby decreasing the anodic current density, which suppresses zinc dendrite growth and enhances cycle performance. The increased rate capability with 3D-Ti-GE can be attributed to the increased active surface area and enhanced kinetics from the resulting electrolyte diffusion pathways. These improvements indicate that this novel electrode configuration is a suitable design for fabricating high-performance ZBBs.

## Methods

### Single cell preparation

The ZBB single cell consisted of the electrolyte, electrodes, bipolar plates (BPs), an ion exchange membrane, tanks, and pumps. The electrolytes were prepared by dissolving 3 M KCl (99.0%, Samchun Chemical, Korea) in a 2.25 M ZnBr_2_ solution (Hanchang Co., Ltd, Korea). The volume of electrolyte was 70 mL on each side. Carbon paper (SIGRACET GDL 38AA, SGL Group, Germany) and graphite plate (SGL Group, Germany) were used as the positive and negative electrodes, respectively, with active areas of 25 cm^2^ (5 cm × 5 cm) each. The Nafion membrane (115, DuPont, Inc., USA) and general graphite BP were used in the cell test. Titanium mesh (Φ0.5 mm, 10 mesh, Nilaco Corporation, Japan) was introduced as the additional electrode, located between the anode and the membrane. For the control unit cell, polyethylene mesh was used as the spacer to introduce electrolyte flow channels. A potentiostat/galvanostat (EC-Lab, Bio-Logic, France) was used to obtain the electrochemical characteristics of the single cell. The cells were cycled in the 0.3–2.8 V range at a current density of 40 mA cm^−2^. The cells were charged for 20 mAh cm^-2^ and then discharged until they reached the cut-off voltage. Using peristaltic pumps (520S, Watson-Marlow), 70 mL of each electrolyte was circulated through the unit cell at a flow rate of 12 mL cm^-2^ min^−1^. In a refreshing process, after every fifth cycle, additional discharge process for zinc stripping was operated with 5 mA cm^−2^, 2 mA cm^−2^, and 1 mA cm^−2^ current density successively until the voltage reached to 0 V. The rate capabilities of the ZBB were investigated at current densities varying from 20 to 80 mA cm^−2^.

### Materials and electrochemical characterization

The anode morphology after successive cycling with and without the 3D electrode structure was investigated using field-emission scanning electron microscopy (FE-SEM) (JSM-7000F, JEOL, Japan). CV profiles were obtained using a typical three-electrode cell. Titanium foil with a geometric area of 2 cm^2^ was used as the working electrode. A saturated calomel electrode (SCE) and platinum electrode were used as the reference and counter electrode, respectively. Structural information of the zinc electrodeposited on the anode was obtained via XRD measurements using an Empyrean diffractometer (PANalytical) equipped with monochromated Cu Kα radiation (λ = 1.54056 Å).
